# Modular Assembly of a Pd Catalyst within a DNA Scaffold for the Amplified Colorimetric and Fluorimetric Detection of Nucleic Acids**

**DOI:** 10.1002/anie.201206006

**Published:** 2012-10-17

**Authors:** Deepak K Prusty, Minseok Kwak, Jur Wildeman, Andreas Herrmann

**Affiliations:** University of Groningen, Zernike Institute for Advanced Materials, Department of Polymer ChemistryNijenborgh 4, 9747 AG Groningen (Netherlands) E-mail: a.herrmann@rug.nl

**Keywords:** bodipy, DNA catalyst, DNA sensing, fluorogenic reaction, signal amplification

Catalytic signal amplification is a powerful tool for the detection of chemical and biological analytes. In chemistry it has been employed in sensing various toxic metal ions (e.g. Pd^2+^, Pb^2+^, Cu^2+^, and Hg^2+^)[1] as well as small molecules such as carbon monoxide[2] and thiols.[3] In a biological context, catalytic reactions have enabled the highly sensitive detection of scarce analytes. They have been widely utilized, for instance, in the detection and assay of proteins,[4] antibodies,[5] and nucleic acids.[6]

In the case of nucleic acids, DNA-templated catalytic processes in particular have been successful. Fluorogenic transformations of this type have been exploited for the amplified detection of deoxyribonucleotides (ODNs) both in homogeneous solutions[7] and in living cells.[8] In this approach, DNA probes are labeled with poorly fluorescent precursors and assembled with target nucleic acids into catalytic hybrids. These then chemically convert the precursors into fluorescent reporters[9] (e.g. through the Staudinger reaction,[10] transthioesterification,[11] and aminolysis).[12] The turnover rate and detection signal can be further improved by repeated thermal cycling. Besides variation of temperature, another external stimulus for signal amplification is light. Photochemical reactions have been employed to trigger the photocatalytic formation of singlet oxygen for the generation of fluorescent reporters.[13] While such systems have resulted in the amplified detection of DNA, RNA, and peptide nucleic acids (PNAs), the approach remains limited by hurdles such as the covalent attachment of profluorescent molecules or photosensitizers to probe ODNs and the need for external stimuli to achieve multiple turnovers.

A potential solution to these drawbacks is grounded in the use of DNA as a structural component, rather than an analyte, in catalytic systems. ODNs on their own are versatile components for catalysis, able to adopt complex three-dimensional structures to catalyze DNA/RNA ligation,[14] DNA phosphorylation,[15] and the formation of nucleopeptide linkages.[16] The same structural properties allow ODNs to serve as effective scaffolds for complex formation with transition metals to form hybrid catalytic systems; Michael addition,[17] Friedel–Craft alkylation,[18] ester hydrolysis,[19] and an enantioselective Diels–Alder reaction[20] have been demonstrated using this concept.

Here we apply the concept of DNA-directed transition-metal catalysts in an entirely new strategy for catalytic signal amplification. We use ligand-labeled probe strands to form a palladium complex in the presence of a specific target DNA sequence. The catalytic center formed on the double-stranded (ds) DNA then efficiently converts water-soluble profluorescent iodo dyes, present in excess, into highly emissive deiodinated reporters. Additionally, since neither the precursor nor the reporter is covalently attached to DNA, no external trigger is required to amplify the fluorescence signal: deiodination of the precursor upon diffusion to the catalytic site is sufficient. This simple DNA-directed catalyst assembly opens the possibility of generating multiple fluorescence signals per hybridization event.

Boron dipyrromethane (BODIPY) derivatives were selected as chromophores because of their 1) high fluorescence quantum yield (FQY), 2) high extinction coefficient, and 3) photostability.[21] We investigated mono- and diiodinated BODIPY precursor chromophores (**1** and **2**, respectively; see Figure 1 a). To ensure water solubility, which is essential for applications in biological systems, a precursor was modified with four triethylene glycol chains. Both profluorescent compounds **1** and **2** proved highly soluble in aqueous media (>10 mg mL^−1^) and were synthesized in 10 and 30 % overall yield, respectively (see pp. 2–3 in the Supporting Information for synthetic details and structural characterization). In both compounds, iodine atoms were incorporated at the C2 and/or C6 positions of the BODIPY core, to favor intersystem crossing to the triplet manifold.[22] The photophysical properties of the precursors and their deiodinated products (**3** and **4**) were initially investigated to confirm their suitability as substrates for fluorogenic reactions in water. Compounds **1** and **2** were individually subjected to palladium-catalyzed deiodination by dissolution in sodium acetate (NaOAc) buffer (0.5 m, pH 5.0) in the presence of a water-soluble Pd catalyst (Na_2_PdCl_4_⋅TPPTS; see pp. 2–3 in the Supporting Information for details). The UV/Vis absorption and fluorescence spectra of the products (**3** and **4**) obtained after 4 h of shaking at room temperature differed markedly from those of the corresponding precursors. As anticipated, the absorption maxima of the dehalogenated products were strongly blue-shifted (Figure 1 b; Figures S1 and S2). Indeed, the color change of the aqueous solution from red to yellow upon deiodination was evident to the naked eye, which implies the possibility for colorimetric detection (similar to Figure 2 c). The fluorescence emission maxima of **3** and **4** also exhibited a blue shift, with fluorescence intensities increasing 35- and 80-fold, respectively, relative to the profluorescent substrates. The significant difference in the fluorescence intensity increase was anticipated in light of the higher background intensity of monoiodo precursor **1**, which we attribute to its having fewer heavy atoms than the diiodo precursor **2**. Moreover, the fluorescence quantum yields (*Φ*_fl_) of fluorophore **3** (*Φ*_fl3_=0.68) and **4** (*Φ*_fl4_=0.81) were 22 and 40 times greater than those of their precursors (Table S1). At this stage, it is important to note that the fluorogenic reaction does not proceed in the absence of any of three reaction components: phosphine ligands and Pd, which form the catalytic complex, and iodo-BODIPY.

**Figure 1:**
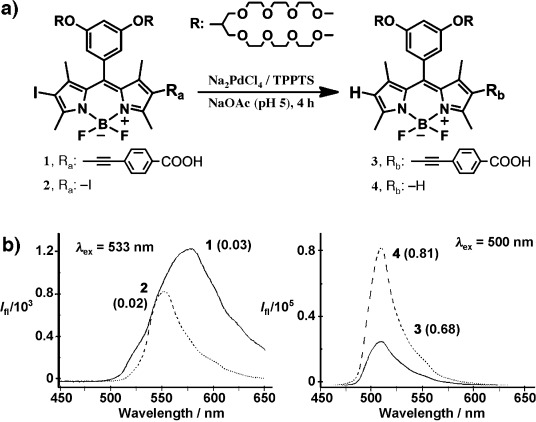
Chemical structures of the water-soluble BODIPY chromophores and their fluorescence spectra. a) Pd-catalyzed dehalogenation of profluorescent mono-/diiodinated precursors (**1** and **2**) to give the fluorescent deiodinated reporters (**3** and **4**). b) Fluorescence spectra of the mono (solid line) and diiodo (dashed line) precursors (left) and the corresponding reporters (right). Numbers in brackets indicate the FQY (*Φ*_fl_) of each compound, as determined against a cresyl violet reference in methanol. See p. 5 in the Supporting Information for additional photophysical properties. TPPTS=sodium triphenylphosphinetrisulfonate.

The suitability of this fluorogenic deiodination reaction for DNA detection was tested with a DNA-templated catalyst (Figure 2 a). Triphenylphosphine ligands were individually coupled through amide bonds to the 5′-end of probe **L** and 3′-end of probe **R** (see Scheme S4 and p. 7 in the Supporting Information for details on the purification and characterization). After HPLC purification, the phosphine-labeled probes were annealed with the target strand (or template, **T**) in a hybridization buffer (see p. 8 in the Supporting Information). Subsequent addition of Na_2_PdCl_4_ with the reducing agent NaBH_4_ resulted in an active catalytic complex owing to the close proximity of the ligands attached to **L** and **R**. Low probe concentrations (≤1 μm) were chosen so that Pd complex formation would only occur through hybridization to the template. It should be noted that in initial experiments we investigated different templates **T** with various nucleotide gaps (0–4 nt) between the two annealing sites of **L** and **R**. When the two probes were separated by 0, 1, and 2 nt an active catalyst was formed; at greater distances (3 and 4 nt) dehalogenation did not occur (data not shown). Since the highest activity was achieved with a single nucleotide gap (see Figure 2 a), this hybrid catalyst in combination with profluorescent BODIPYs was used to detect the presence of a target DNA sequence, which we investigated in terms of reaction kinetics, scope, and detection limit.

**Figure 2:**
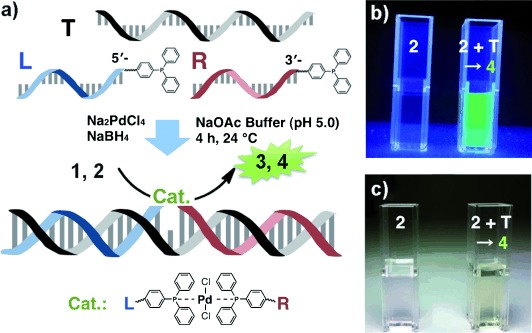
DNA-directed assembly of the catalyst and subsequent generation of the fluorophore. a) Pd-catalyzed detection of the target sequence (**T**, 30-mer) mediated by the complexation of phosphine-modified ODN probes **L** and **R** (15-mer and 14-mer, respectively) with Pd to form the Cat. site. b,c) Photographs of reaction mixtures (precursor **2**, **L**, **R**, Na_2_PdCl_4_, and NaBH_4_ in NaOAc buffer) with (right) and without (left) **T** (1 nm) taken under UV (365 nm) exposure (b) and ambient light (c). In the presence of the template, conversion of **2** to **4** resulted in a clear color and fluorescence emission.

The DNA-directed catalyst for the fluorogenic reaction was tested at a range of concentrations of the target strand **T** (from 1 nm to 1 fM) and iodo-BODIPY **1** or **2**, with fixed concentrations of the probes **R** and **L**, and catalyst Na_2_PdCl_4_, and NaBH_4_ (see p. 8 in the Supporting Information). The reactions were performed in NaOAc buffer at pH 5 under inert conditions. It is noteworthy that the reaction required an acidic pH probably to prevent complexation of Pd with the nucleobases. After gentle mixing for 4 h, the reaction mixtures exhibited the expected intense fluorescence resulting from multiple turnovers of the deiodination reaction (Figure 2 b). As negative controls, all reactions were also performed without the template or catalyst, or with a template containing a single-base mismatch (**T-sbm**). When the fully complementary template was used, 90 % of the fluorescence maximum was reached after 4 min for a template concentration of 1 nm, and saturation was achieved within 10 min (Figure 3 a, curve 1). This rate of reaction is twice that of a DNA-templated Heck reaction used to deiodinate an analogous BODIPY-DNA conjugate.[22a] A possible explanation for this improvement is that the dehalogenation reaction entails fewer intermediates than the Heck cross-coupling.[23] It should be also noted that at lower template concentrations (100 pm and 10 pm) the reaction is so rapid (Figure 3 a, curves 2 and 3) that detection assays could be performed within only several minutes. In contrast, the use of **T-sbm** in identical conditions slowed the reaction dramatically, reducing the initial increase in intensity by 65-fold (Figure 3 a, curve 4). Comparing this finding to an analogous study using the Pd-catalyzed C–C cross-coupling (55-fold slowing),[22a] we see a greater degree of sequence selectivity using the DNA hybrid catalyst.

**Figure 3:**
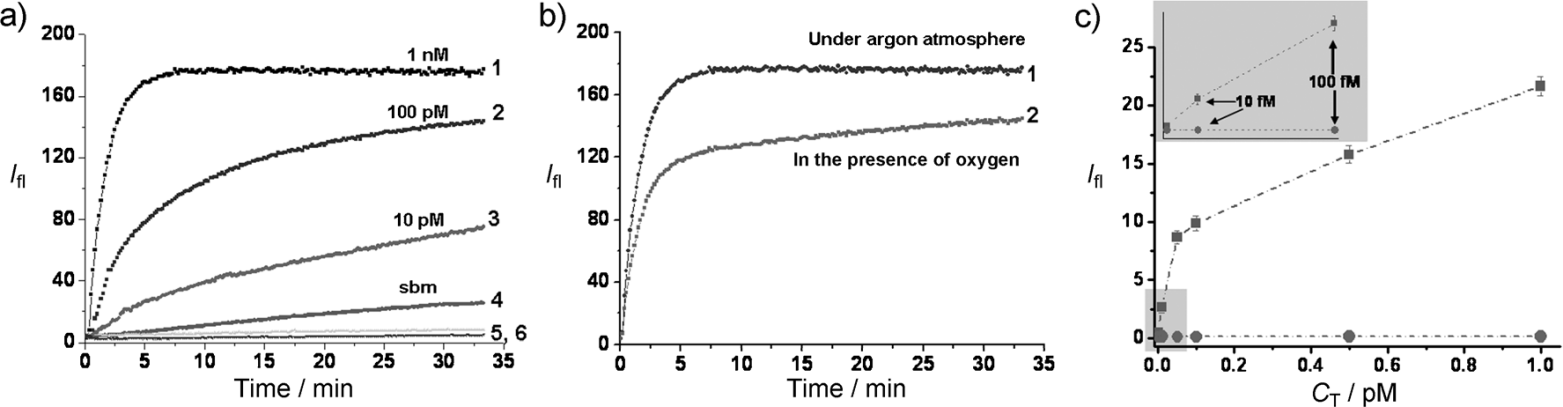
Fluorimetric determination of the reaction kinetics and the limit of detection of the catalytic conversion. a) Evolution of fluorescence intensity over time at target concentrations (*C*_T_) of 1 nm to 10 pm (curves 1–3), in the presence of **T-sbm** with catalyst (curve 4), in the presence of **T** without catalyst (curve 5), and without **T** (curve 6) for the conversion of **1** into **3**. b) Comparison of the reaction kinetics for the conversion of **1** into **3** under an argon atmosphere (curve 1) and under ambient air (curve 2) in the presence of *C*_T_=1 nm and 10 nm monoiodo substrate. c) Fluorescence intensities corresponding to the the conversion of **1** into **3** for *C*_T_=1, 10, 100, 500, and 1000 fm (squares) and without template (circles) at a fixed concentration of the monoiodo substrate of 500 fm. Magnified sub-picomolar range of the graph (inset). All fluorogenic dehalogenations were monitored at 510 nm (*λ*_ex_=500 nm).

In further experiments we investigated the scope and utility of the DNA-mediated Pd-catalyzed dehalogenation reaction. Since an inert gas atmosphere complicates the applicability of an analytical assay we performed the catalysis in the presence of air. As can be seen from Figure 3 b, the presence of oxygen affects the catalysis; however, the initial rates for the two reactions are very similar, indicating that the transformation still proceeds at a fast rate even in oxygenated environments. In the next step, we performed the catalytic dehalogenation assay in more complex environments. We studied the performance of the catalyst in the presence of crude cell extract and proteins (bovine serum albumine (BSA) and DNA polymerase) (see p. 10 in the Supporting Information). The results indicate that the catalyst remains active in crude cell extract and in the presence of the polymerase; however, it loses its activity when BSA is present (Figure S6). It is not surprising that no activity was detected for the sample containing BSA. This protein, which contains 35 cysteines, was applied in twofold excess relative to Pd and it is well known that thiols are toxic for palladium catalysts. From these measurements it can be concluded that the DNA-mediated Pd catalyst system is robust enough for practical sensing applications.

In terms of sensitivity, we investigated both colorimetric and fluorimetric limits of detection. A series of reaction mixtures with differing target concentrations and a fixed concentration of **2** (0.1 μm) was incubated for 4 h, and a clear transition from red to yellow could be observed with the naked eye for target concentrations down to 1 nm (Figure 2 c). This visual ODN detection limit in our DNA-templated system is somewhat limited compared to that of other systems, such as plasmonic enhancement of inorganic particles[24] and the DNA-photograph technique,[25] because the observation here depends solely upon the absorption shift of the iodinated versus the deiodinated BODIPY from *λ*_max_=533 to 500 nm (Figure S1a in the Supporting Information) However, the amplified conversion to reporter dyes in our system and the high extinction coefficient of BODIPY analogues made such simple and immediate visual detection possible without any additional instruments.

When fluorescence rather than the relatively small absorption shift is used as the sensing parameter, the detection limit improves by up to 5 orders of magnitude. This limit of detection was defined as the target concentration at which the fluorescence signal could be distinguished from that of the corresponding negative control without the template, namely *C*_T_=100 fm with diiodo-BODIPY **2** and 10 fm using monoiodo-BODIPY **1** as the chromophore precursors (see p. 11 in the Supporting Information). The difference in the detection limits for the two compounds is a simple consequence of number of iodine atoms in the precursors. Below a target ODN concentration of 100 fm, the catalytic complex can still quite efficiently remove single iodine atoms from the abundant profluorescent substrates, yielding a strong signal for the conversion of **1** to **3**. However, precursor **2** must be deiodinated twice to produce a significant fluorescence signal, and at such low concentrations of the catalytic complex the predominant product is monohalogenated **5** (Scheme S3), which has a low FQY (*Φ*_fl5_=0.03) and exhibits a much smaller blue shift in both fluorescence and absorption (Figure S3). The different conversion capabilities of the mono and diiodo precursors at low target concentrations were investigated further in terms of the quantitative threshold for complete fluorogenic conversion as a function of *C*_T_ (see p. 12 in the Supporting Information). The lower conversion efficiency for diiodo precursor **2** (Figure 4) at all tested target concentrations is consistent with a single deiodination event per diiodo molecule. This result merits further investigation to better understand catalytic conversions at low concentrations. Single-molecule spectroscopy may be a suitable tool to study the efficient and sensitive fluorogenic catalytic conversion presented here in greater depth, as using DNA in our catalytic model offers several advantages: facile functionalization, immobilization, and sequence-specific assemblies.

**Figure 4:**
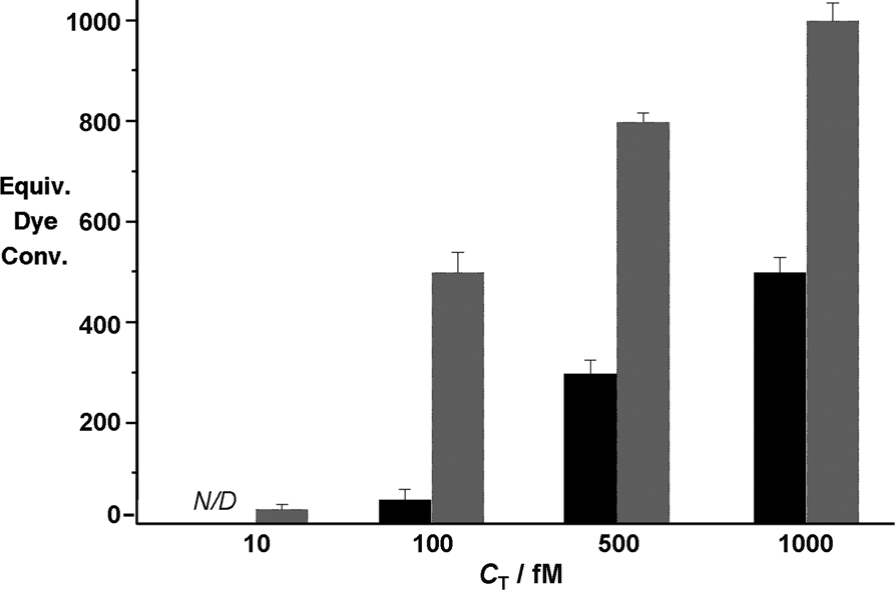
Equivalents of di-/monoiodo (black/gray) dye molecules converted per target molecule. Each threshold was determined by the complete conversion of the dye added in various amounts (30–100 equiv) at fixed *C*_T_=10, 100, 500, and 1000 fm. At *C*_T_=10 fm, complete conversion of diiodo-BODIPY **2** could not be detected at any dye concentration (*N/D*). All fluorogenic dehalogenations were monitored at 510 nm (*λ*_ex_=500 nm).

In summary, we have developed a novel and modular DNA–transition-metal hybrid catalyst based on the powerful class of Pd-catalyzed reactions. At the same time a concept for amplified DNA detection combining iodo-BODIPYs with the DNA–Pd complex was successfully realized, allowing the generation of multiple signals from a single hybridization event. This simple assay is suitable for rapid colorimetric or fluorimetric detection of nucleic acid targets. Phosphine ligands for the catalytic complex, rather than dye precursors, are conjugated directly to the DNA probes, yielding an active catalytic complex upon hybridization to the target sequence. As such, each hybridization event can catalyze the fluorogenic conversion of many precursor dye molecules, producing hundreds of fluorophores even in sub-picomolar target concentration. This is an obvious improvement over the standard method of conjugating reporter dye precursors to the probes, which is limited to one fluorophore per target hybridization. The result of this strategy is the amplified ultrasensitive detection of DNA down to a limit of 10 fm, to the best of our knowledge 2 orders of magnitude better than that reported for any other DNA-templated fluorogenic reaction.[22a] Furthermore, this sequence-dependent construction of the catalyst to convert fluorogenic substrates present in solution is a powerful alternative to other DNA-sensing methods, particularly since the dissociation of the probe from the target is not necessary to achieve turnover and fluorescent signal amplification. Besides, since our method requires no complex design or external stimuli, it should be equally applicable to other DNA or RNA structures like aptamers for the sensing of other biomolecules and small-molecule analytes.

## Supplementary material

Detailed facts of importance to specialist readers are published as ”Supporting Information”. Such documents are peer-reviewed, but not copy-edited or typeset. They are made available as submitted by the authors.
