# Supplementary material for: Modular Assembly of a Pd Catalyst within a DNA Scaffold for the Amplified Colorimetric and Fluorimetric Detection of Nucleic Acids
Source: Angew Chem Int Ed Engl. 2012 Oct 17;51(47):11894–8. doi: 10.1002/anie.201206006 (PMC3533772; doi:10.1002/anie.201206006)
Supplement: Supplementary file 1 [file anie0051-11894-SD1.pdf]

Angewandte Chemie
Eine Zeitschrift der Gesellschaft Deutscher Chemiker

Supporting Information

© Wiley-VCH 2012

69451 Weinheim, Germany

# Modular Assembly of a Pd Catalyst within a DNA Scaffold for the Amplified Colorimetric and Fluorimetric Detection of Nucleic Acids**

*Deepak K. Prusty, Minseok Kwak, Jur Wildeman, and Andreas Herrmann**

anie_201206006_sm_miscellaneous_information.pdf

Appendices

1. Materials and Methods....................................................................S2

2. Synthesis of Water-Soluble Bisiodinated BODIPY Substrate........................S3-S5

3. Synthesis of Water-Soluble Monoiodinated BODIPY Substrate.....................S6-S7

4. Palladium-Catalyzed Deiodination of BODIPY Substrates...............................S7

5. Photophysical Properties of BODIPY Substrates and Reporter Dyes............S8-S9

6. ODN Synthesis and Characterization..............................................S10

7. Synthesis and Characterization of $PPh_3$-Labeled ODN Conjugates............S11-S13

8. Conditions for DNA-Templated Deiodination Reaction..................................S13

9. Kinetics of DNA-Templated Deiodination Reaction.......................................S14

10. Pd-Catalyzed Dehalogenation Assay in Presence of Crude Extract..................S14

11. Determination of Limit of Detection..................................................S15

12. Determination of Quantitative Conversion Thresholds..................................S15

# 1. Materials and Methods

All chemicals and reagents were purchased from commercial suppliers and used without further purification, unless otherwise noted. The 3,5-dihydroxybenzaldehyde (98%), 2,4-dimethylpyrrole (95%), 2,3-dichloro-5,6-dicyano-1,4-benzoquinone (DDQ, 98%) trifluoroacetic acid (99%), iodic acid (99.5%), iodine (99.99%), tetrabutylammonium iodide (n-Bu4NI, 99%), CuI (99.5%), N-hydroxy-succinimide (NHS, 98%), tri-*tert*-butylphosphine ($P(t\text{-}Bu)_3$, 98%), tris(dibenzylideneacetone)dipalladium(0) ($Pd_2(dba)_3$), sodium-tetrachloro-palladate(II) ($Na_2PdCl_4$, 99.99%), 1,4-dioxane (99%), triphenylphosphine carboxylic acid (98%), *N,N'*-dicyclohexylmethylamine ($CY_2NMe$, 97%), and dimethylformamide (99%) were purchased from Sigma-Aldrich and used as received. Other special chemicals obtained from different chemical sources were tris(3-sulfonatophenyl)phosphine hydrate sodium salt ($P(p\text{-}SO_3C_6H_4Na)_3$, Strem Chemicals), 4-ethynylbenzoic acid (96%, ChemBridge Corporation) and *N,N'*-dicyclohexyl-carbodiimide (99%, Merck). Both modified and unmodified oligonucleotides (ODNs) were synthesized using standard automated solid-phase phosphoramidite coupling methods on an ÄKTA oligopilot plus (GE Healthcare) DNA synthesizer. All solvents and reagents for oligonucleotide synthesis were purchased from Novabiochem (Merck, UK) and SAFC (Sigma-Aldrich, Netherlands). Solid supports (Primer SupportTM, 200 μmol/g) from GE Healthcare were used for the synthesis of DNA. Oligonucleotides were purified by reverse-phase High Pressure Liquid Chromatography (HPLC) using a C15 RESOURCE RPC™ 1 mL reverse phase column (GE Healthcare) through custom gradients using elution buffers (A: 100 mM triethylammonium acetate (TEAAc) and 2.5% acetonitrile and B: 100 mM TEAAc and 65% acetonitrile). Fractions were further desalted by either desalting column (HiTrapTM desalting, GE Healthcare) or dialysis membrane (MWCO 2000, Spectrum® Laboratories). Labeled oligonucleotides were purified by HPLC and characterized by MALDI-TOF mass spectrometry using a 3-hydroxypicolinic acid matrix. The spectra were recorded on an ABI Voyager DE-PRO MALDI TOF (delayed extraction reflector) Biospectrometry Workstation mass spectrometer. $^{1}$H-NMR and $^{13}$C-NMR spectra were recorded on a Varian Mercury (400 MHz) NMR spectrometer at 25 $^{o}$C. High-resolution mass spectra (HRMS) were recorded on an AEI MS-902 (EI+) instrument. Absorption and fluorescence spectra of both the non-templated and templated products of the fluorogenic reactions and the concentration of the DNA were measured on a SpectraMax M2 spectrophotometer (Molecular Devices, USA) using 1 cm light-path quartz cuvette. Column chromatography was performed using silica gel 60 Å (200-400 Mesh).

## 2. Synthesis of Water-Soluble Bisiodinated BODIPY Substrate

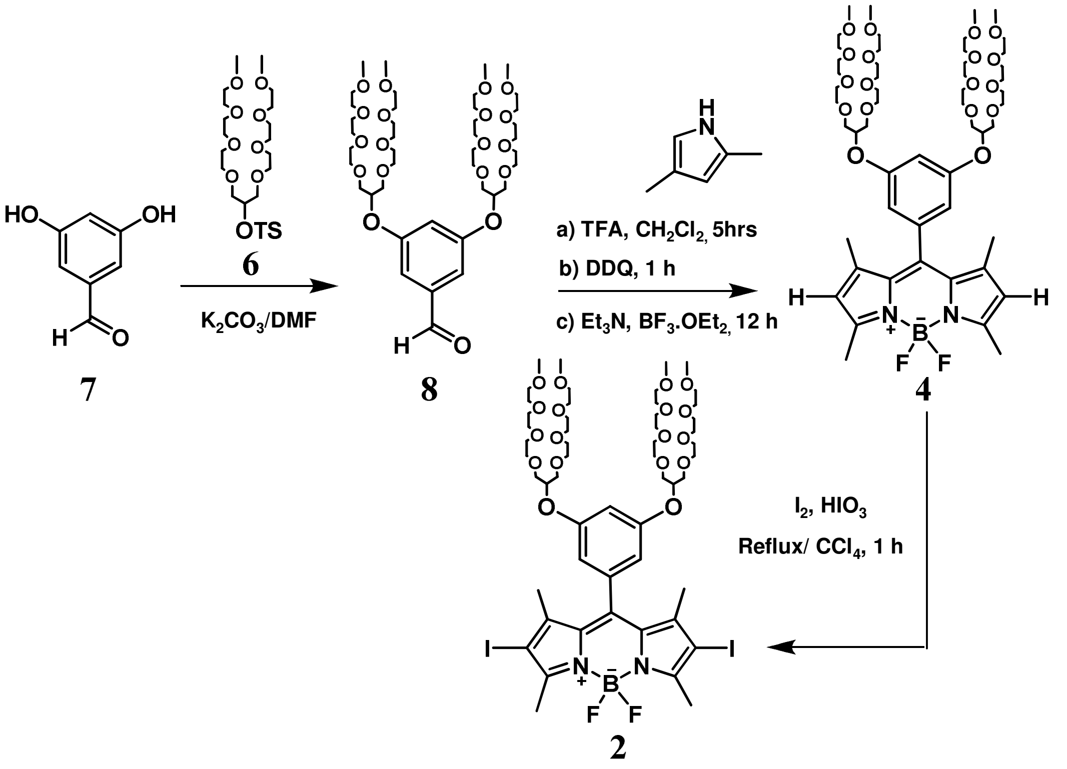

**Scheme S1**. Synthetic route to the oligoethylene-modified bisiodinated BODIPY precursor.

### *2.1. 3,5-di[1-(1',3'-bis-(3',6',9'-trioxadecylglyceryl]benzaldehyde (8)*

The starting material 3,5-dihydroxybenzaldehyde (**7**) was received from a commercial source and 1,3-bis(3,6,9-trioxadecanyl) glycerol-2-toluenesulfonic ester (**6**) was synthesized as reported elsewhere.[1] Compound **6** (16.4 g, 30.4 mmol) and $K_2CO_3$ (5.6 g, 40.6 mmol) were added to a solution of compound **7** (2.0 g, 14.5 mmol) in dry DMF (10 mL). The mixture was stirred at 65 °C under continuous nitrogen atmosphere for 48 h. The progress of the reaction was monitored by TLC. The reaction mixture was cooled down to RT and a mixture of water (5 mL) and brine (5 mL) was added. The resulting solution was extracted with $CHCl_3$ (3 × 20 mL) and the combined organic layers were further washed with brine (3 × 10 mL). The resulting crude mixture was purified by silica gel column chromatography using EtOAc/$CH_2Cl_2$/MeOH (5:10:2 v/v) to yield compound **8** as a colorless oil (1.1 g, 75%).

**$^1$H NMR (400 MHz, $CDCl_3$) δ (ppm)**: 3.48 (s, 12H), 3.49-3.66 (m, 56H), 4.43-4.46 (m, 2H), 6.82 (s, 1H), 7.12 (s, 2H), 9.83 (s, 1H).
**$^{13}$C NMR (100 MHz, $CDCl_3$) δ(ppm)**: 59.21, 70.55, 70.65, 70.68, 70.78, 70.79, 111.0, 111.9, 130.1, 154.7, 191.2.
**AEI MS-902 (EI+)**: Calculated mass for $C_{41}H_{74}O_{19}$ $[M+H]^+$: 870.48; found: 870.52.
**Elemental analysis**: Anal. calculated for $C_{41}H_{74}O_{19}$: C, 56.54; H, 8.56; found: C, 56.49; H, 8.58.

### *2.2. 4,4-Difluoro-8-{3,5-di[1-(1',3'-bis-(3',6',9'-trioxadecylglyceryl]}benzaldehyde-1,3,5,7-tetramethyl-4-bora-3a,4a-diaza-s-indacene (4)*

Compound **5** (2.61 g, 3.0 mmol) and 2,4-dimethylpyrrole (0.571 g, 6.0 mmol) were dissolved in dry $CH_2Cl_2$ (50 mL) under nitrogen atmosphere. Three drops of trifluoroacetic acid (TFA) were added and the resulting reaction mixture was stirred at room temperature in the dark for 5 h. A solution of 2,3-dichloro-5,6-dicyano-1,4-benzoquinone (1.36 g, 6.0 mmol) in dry $CH_2Cl_2$ (5 mL) was added dropwise to the reaction mixture. This reaction mixture was stirred for an additional 1 h at room temperature. Subsequently, freshly distilled borontrifluoride diethyl etherate ($BF_3·OEt_2$, 15 mL) was added at 5 °C, followed by triethylamine (15 mL). The reaction mixture was stirred at room temperature overnight and then concentrated under reduced pressure, dissolved in $CH_2Cl_2$, and washed with water (3 × 20 mL). The organic layer was further washed with brine (20 mL), followed by drying over $Na_2SO_4$ and concentration *in vacuo.* The crude product was purified twice by silica gel column chromatography using $CHCl_3$/ MeOH (100:2) and $CHCl_3$/ THF (10:3) as mobile phase, yielding compound **4** as an orange oil with bright green fluorescence (1.48 g, 45%).

**$^1$H NMR (400 MHz, $CDCl_3$) δ(ppm)**:1.51 (s, 6H), 2.51 (s, 6H), 3.34 (s,12H), 3.48-3.71 (m, 56H), 4.43-4.46 (m, 2H), 5.94 (s, 2H), 6.52 (s, 2H), 6.64 (s, 1H).
**$^{13}$C NMR (100 MHz, $CDCl_3$) δ(ppm)**:14.55, 14.77, 59.21, 70.55, 70.65, 70.68, 70.78, 70.79, 106.21, 108.93, 121.31, 131.25, 136.69, 141.48, 143.17, 155.66, 160.52.
**AEI MS-902 (ESI+)**: Calculated mass for $C_{53}H_{87}N_2O_{18}BF_2$ $[M+Na]^+$: 1112.07; found: 1111.59.
**Elemental analysis**: Anal. calculated for $C_{53}H_{87}N_2O_{18}BF_2$: C, 58.45; H, 8.05; N, 2.57; found: C, 58.44; H, 8.12; N, 2.49.

### *2.3. 4,4-Difluoro-8-{3,5-di[1-(1',3'-bis-(3',6',9'-trioxadecylglyceryl]}benzaldehyde-1,3,5,7-tetramethyl-2,6-diiodo-4-bora-3a,4a-diaza-s-indacene (2)*

Iodic acid ($HIO_3$, 1.2 g, 6.8 mmol) in water (7 mL) was added dropwise to a solution of compound **2** (3.37 g, 3.1 mmol) and iodine (1.0 g, 7.8 mmol) in ethanol (30 mL) over 10 min. After the addition was complete, the mixture was stirred for an additional 1 h. The progress of the reaction was followed by TLC until completion. Ethanol was removed *in vacuo* and the remaining aqueous solution was extracted with dichloromethane. The organic layer was dried over anhydrous $Na_2SO_4$ and concentrated under reduced pressure. The crude mixture was further purified by silica gel column chromatography using $CH_2Cl_2$ as mobile phase yielding compound **2** as a red solid (3.5 g, 85%).

**$^1$H NMR (400 MHz, $CDCl_3$) δ(ppm)**:1.52 (s, 6H), 2.6 (s, 6H), 3.33 (s,12H), 3.48-3.66 (m, 56H), 4.45-4.47 (m, 2H), 6.49 (s, 2H), 6.7 (s, 1H).

**$^{13}$C NMR (100 MHz, $CDCl_3$) δ(ppm)**:16.18, 17.11, 59.19, 70.19, 70.59, 70.64, 70.67, 70.77, 72.08, 85.71, 106.45, 108.59, 131.12, 132.98, 136.26, 141.19, 145.41, 160.81.

**AEI MS-902 (ESI+)**: Calculated mass for $C_{53}H_{85}N_2O_{18}I_2BF_2$ $[M+Na]^+$: 1363.39; found 1363.38.

**Elemental analysis**: Anal. calculated for $C_{53}H_{85}N_2O_{18}I_2BF_2$: C, 47.47; H, 6.39; N, 2.09; found: C, 47.51; H, 6.37; N, 2.11.

## 3. Synthesis of Water-Soluble Monoiodinated BODIPY Substrate

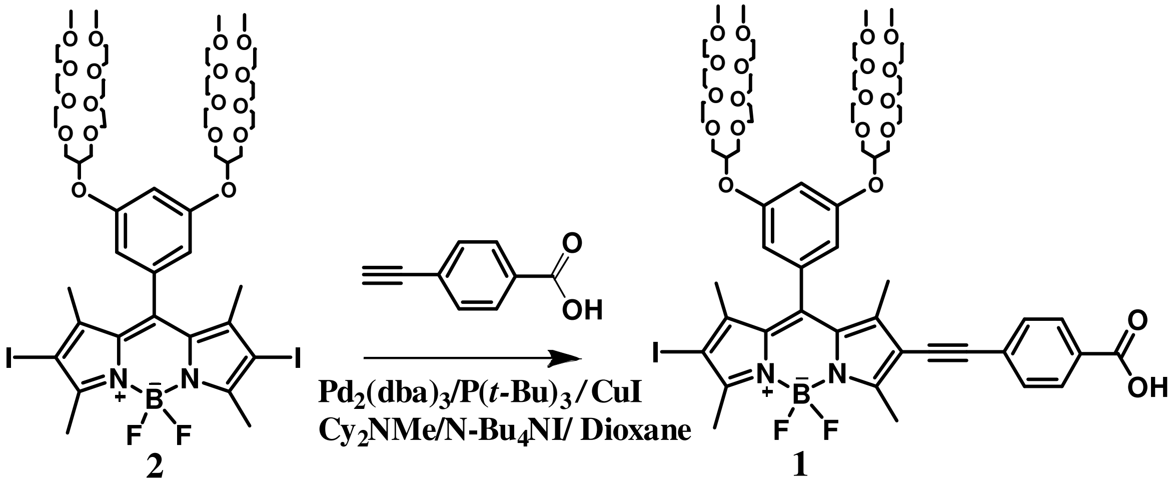


**Scheme S2.** Synthetic route to the oligoethylene-modified carboxyl-functionalized mono-iodinated BODIPY.

$Pd_2(dba)_3$ (7.0 mg , 0.008 mmol), P(*t*-Bu)$_3$ ( 0.033 mmol, 48 μL), n-$Bu_4NI$ (18 mg, 0.05 mmol) and CuI (5.0 mg, 0.026 mmol) were added to a solution of compound **2** (54.0 mg, 0.04 mmol) in dry 1,4-dioxane (2 mL) and *N,N'*-dicyclohexylmethylamine (0.2 mL, 0.94 mmol ) at 24 °C followed by addition of 4-ethynylbenzoic acid (15.0 mg, 0.1 mmol) under argon atmosphere. The reaction mixture was stirred for 2 h at room temperature under continuous argon atmosphere. The progress of the reaction was monitored by TLC. After completion of the reaction, water (10 mL) was added to the reaction mixture. The resulting solution was extracted with $CH_2Cl_2$ (2 × 50 mL), followed by drying over anhydrous $MgSO_4$ and evaporation of the solvent under reduced pressure to obtain the crude product. Silica gel column chromatography using EtOAc/$CHCl_3$/MeOH (10/10/1) as eluent afforded compound **1** as a red solid (12 mg, 25% yield).

**$^1$H NMR (400 MHz, $CDCl_3$) δ(ppm)**:1.52 (s, 6H), 2.6 (s, 6H), 3.33 (s,12H), 3.48-3.66 (m, 56H), 4.46-4.48 (m, 2H), 6.41 (s, 2H), 6.58 (s, 1H), 7.51 (d, 2H, J = 7.42), 8.04 (d, 2H, J = 7.99).

**$^{13}$C NMR (100 MHz, $CDCl_3$) δ(ppm)**:16.18, 17.11, 59.19, 70.19, 70.59, 70.64, 70.67, 70.77, 72.08,85.16, 85.54, 95.82, 102.53, 105.95, 115.43, 128.58, 128.75, 130.06, 131.08, 131.79, 135.72, 141.95,144.75, 145.40, 157.04, 157.96, 161.64, 170.10.

**AEI MS-902 (ESI+)**: Calculated mass for $C_{62}H_{90}N_2O_{20}IBF_2$ $[M+Na]^+$: 1381.52; found: 1381.50. **Elemental analysis**: Anal. calculated for $C_{62}H_{90}N_2O_{20}IBF_2$: C, 54.79; H, 6.67; N, 2.06; found: C, 54.83; H, 6.69; N, 2.03.

## 4. Palladium-Catalyzed Deiodination of Water-Soluble Precursors **1**, **2** to Fluorescent Products **3**, **4** and Mono-Dehalogenated Byproduct **5**

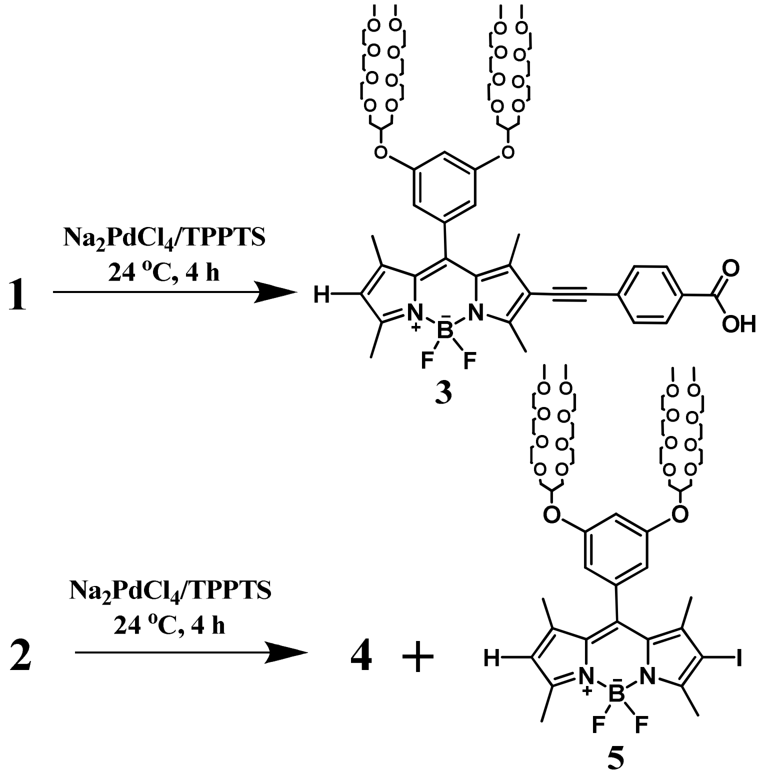


**Scheme S3**. Synthetic route to the deiodinated fluorescent products of water-soluble mono- and bisiodo BODIPY substrates.

To a solution of BODIPY precursor **1** or **2** (100 µM, 80 µL) in sodium acetate buffer (0.5 M, pH = 5.0) in two separate vials 20 µL of water-soluble Pd catalyst (20 µM $Na_2PdCl_4$ and 40 µM $P(p\text{-}SO_3C_6H_4Na)_3$ pre-mixed for 15 min) was added. The resulting 100 µL reaction mixtures were shaken at 24 °C for 4 h. The progress of the reaction was monitored by TLC. Finally, UV/Vis and fluorescence spectra were employed to characterize the fully deiodinated products **3**, **4** and the mono-dehalogenated byproduct **5** of bisiodo precursor **2**.

## 5. Photophysical Properties of BODIPY Substrates and Reporter Dyes

### *5.1 Monoiodinated BODIPY 1 and fluorescent deiodination product 3*

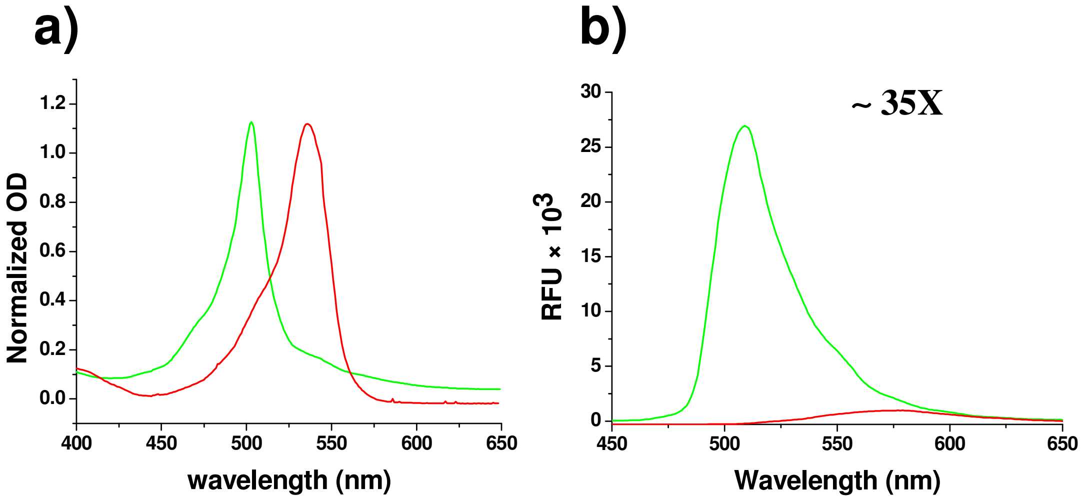


**Figure S1.** (a) Normalized absorption spectra of **1** (red, $\lambda_{max}$ = 533) and **3** (green, $\lambda_{max}$ = 500) in water at 24 °C. (b) Relative fluorescence emission spectra of **1** (red, $\lambda_{em}$ = 577) and **3** (green, $\lambda_{em}$ = 510) in water at 24 °C.

### *5.2 Bisiodinated BODIPY 2 and fluorescent deiodination product 4*

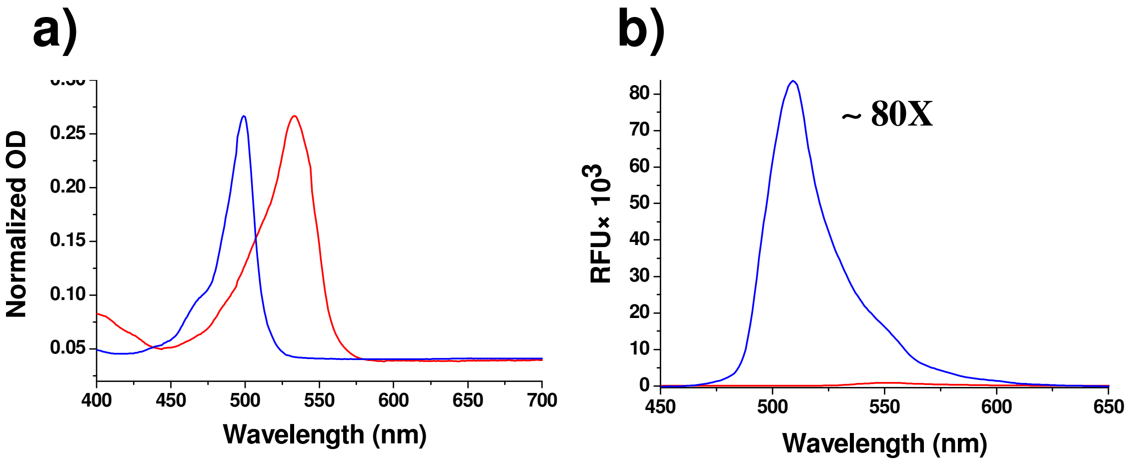


**Figure S2.** (a) Normalized absorption spectra of **2** (red, $\lambda_{max}$ = 533) and **4** (blue, $\lambda_{max}$ = 500) in water at 24 °C. (b) Relative fluorescence emission spectra of **2** (red, $\lambda_{em}$ = 552) and **4** (blue, $\lambda_{em}$ = 510) in water at 24 °C.

### 5.3 *Bisiodinated BODIPY 2 and mono-dehalogenated byproduct 5*

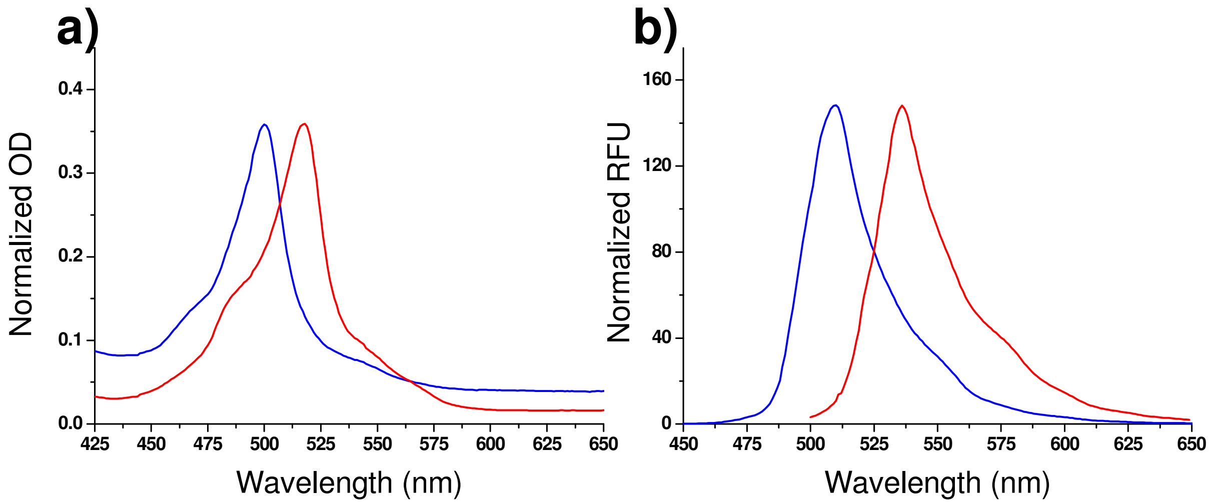


**Figure S3.** (a) Normalized absorption spectra of **2** (blue, $\lambda_{max}$ = 500) and **5** (red, $\lambda_{max}$ = 518) in NaOAc buffer at 24 °C. (b) Normalized fluorescence emission spectra of **2** (blue, $\lambda_{em}$ = 510) and **5** (red, $\lambda_{em}$ = 535) in NaOAc buffer at 24 °C.

**Table S1.** Photophysical properties of the mono- and bisiodinated BODIPY probes **1** and **2** as well as the dehalogenated fluorescent products **3** and **4**.

| Compound | Absorbance $\lambda_{max}$ [nm] | Excitation coefficient [ $M^{-1}$ $cm^{-1}$] | Emission $\lambda_{max}$ [nm] | Fluorescence quantum yield ($\Phi_{fl}$) |
|---|---|---|---|---|
| 1 | 533 | $7.8\times10^{4}$ | 577 | 0.03 |
| 2 | 533 | $9.7\times10^{4}$ | 552 | 0.02 |
| 3 | 500 | $9.5\times10^{4}$ | 510 | 0.68 |
| 4 | 500 | $10.8\times10^{4}$ | 510 | 0.81 |

## 6. ODN Synthesis and Characterization

All oligonucleotides (**Table S2**) were synthesized in 10 µmol scale on an ÄKTA oligopilot plus (GE Healthcare) DNA synthesizer using standard β-cyanoethylphosphoramidite coupling chemistry. Deprotection and cleavage from the PS-support were carried out by incubation in concentrated aqueous ammonium hydroxide solution for 5 h at 55 ℃. Following deprotection, the oligonucleotides were purified by using anion exchange chromatography, HiTrap™ Q HP 1 mL or 5 mL column (GE Healthcare) through custom gradients using elution buffers (A: 25 mM Tris, pH = 8.0, B: 25 mM Tris and 1.0 M NaCl). Fractions were further desalted by either desalting column (HiTrap™ desalting, GE Healthcare) or dialysis membrane (MWCO 2000, Spectrum® Laboratories). Oligonucleotide concentrations were determined by UV absorbance using extinction coefficients. Finally, the identity of the oligonucleotides was confirmed by MALDI-TOF mass spectrometry (**Table S2**).

**Table S2.** Sequences and MALDI-TOF mass spectrometry data of the triphenylphosphine-modified probes [**L**], [**R**] ODNs and target strands **T** and **T-sbm** used for our study.

| ODN | DNA Sequence (5′ to 3′) | Calculated (m/z) | Found (m/z) |
|---|---|---|---|
| **L** | **$NH_2$**-(C6)-TAG TAT ATA TCT TGC-3′ | 4736 | 4734 |
| **R** | 5′-ATC TTT AGT TTA GC-(C7)**$NH_2$** | 4453 | 4453 |
| **T**[a] | 5′-GCA AGA TAT ATA CTA GGC TAA ACT AAA GAT-3′ | 9255 | 9258 |
| **T-sbm**[b] | 5′-GCA AGA TAT ATA GTA GGC TAA ACT AAA GAT-3′ | 9289 | 9291 |

[a]Fully matched and [b]single-base-mismatched (C to G mutation) sequences for T architecture.

# 7. Synthesis and Characterization of Triphenylphosphine ($PPh_3$)-Labeled ODN Conjugates

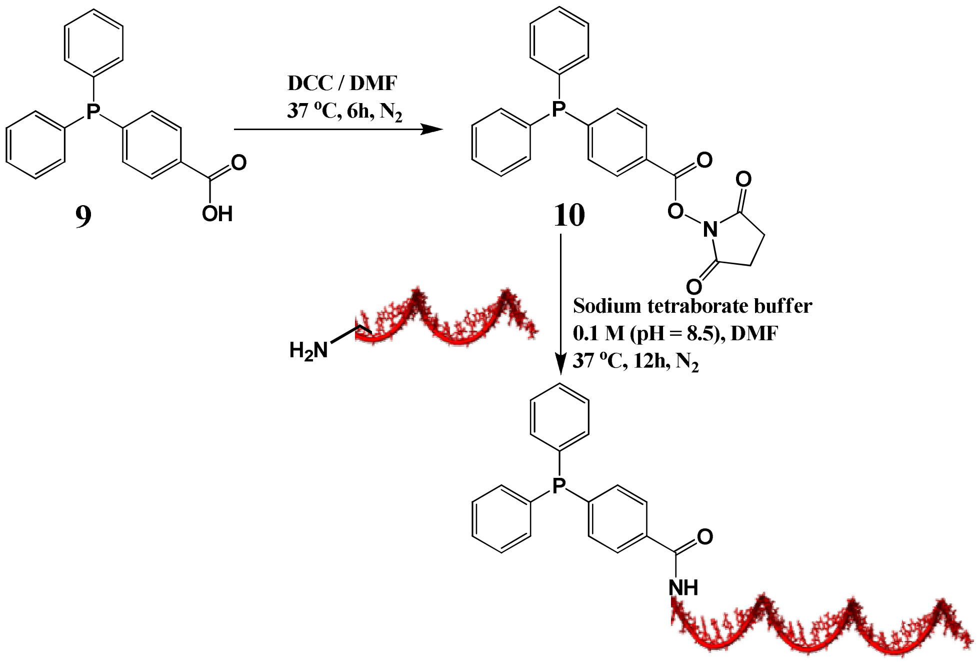


**Scheme S4**. Synthetic route for $PPh_3$-labeled ODN probes (**L** and **R**).

## *7.1 Synthesis of NHS ester of triphenylphosphine ($PPh_3$) lignad*

The carboxyl group of triphenylphosphine (TPP) ligand was activated by reacting compound **9** (0.0306 g, 0.1 mmol) with *N*-hydroxy succinimide (NHS) (0.0364 g, 0.3 mmol) and *N,N'*-dicyclohexyl-carbodiimide (0.037 g, 0.32 mmol) in 2 mL of DMF. The reaction was carried out for 24 h under inert atmosphere at room temperature (**Scheme S4**). Precipitated dicyclohexylurea (DCU) was removed by filtration. The solvent was evaporated under reduced pressure and the crude mixture was purified by column chromatography using hexane/EtOAc (1:1) as eluent. Activated product **10** was obtained as colorless solid (27 mg, 67%).

.

### *7.2 DNA labeling with $PPh_3$-NHS ester*

5′-(C6)-Amino-modified oligonucleotides **L** and **R** (**Table S2**) were dissolved in sodium tetraborate buffer (0.1 M, pH = 8.5) in two separate vials at concentrations of 1 nmol/μL. 100 μL of each amino-modified oligonucleotide solution was reacted separately in two different vials, each containing a solution of activated $PPh_3$–NHS ester **10** in dimethylformamide (20 μL, 40 μg/μL). The resulting reaction mixtures were mixed in a shaker for 24 h at ambient temperature (**Scheme S4**). The reaction mixtures were freeze-dried to remove the DMF-$H_2O$ mixture. Purification of the labeled oligonucleotides was carried out by using reverse-phase HPLC employing a C15 RESOURCE RPC™ 1 mL column (GE Healthcare) through custom gradients using elution buffers (A: 100 mM TEAAc and 2.5% acetonitrile, B: 100 mM TEAAc and 65% acetonitrile). The coupling yield of the labeling reaction was estimated to be 60% from the integration of the peaks of the HPLC chromatogram. The purified $PPh_3$-labeled oligonucleotides **L** and **R** (band at ~20 mL of **Figure S4A** and **S5A**) were analyzed by MALDI-TOF mass spectrometry (**Figure S4B** and **S5B**).

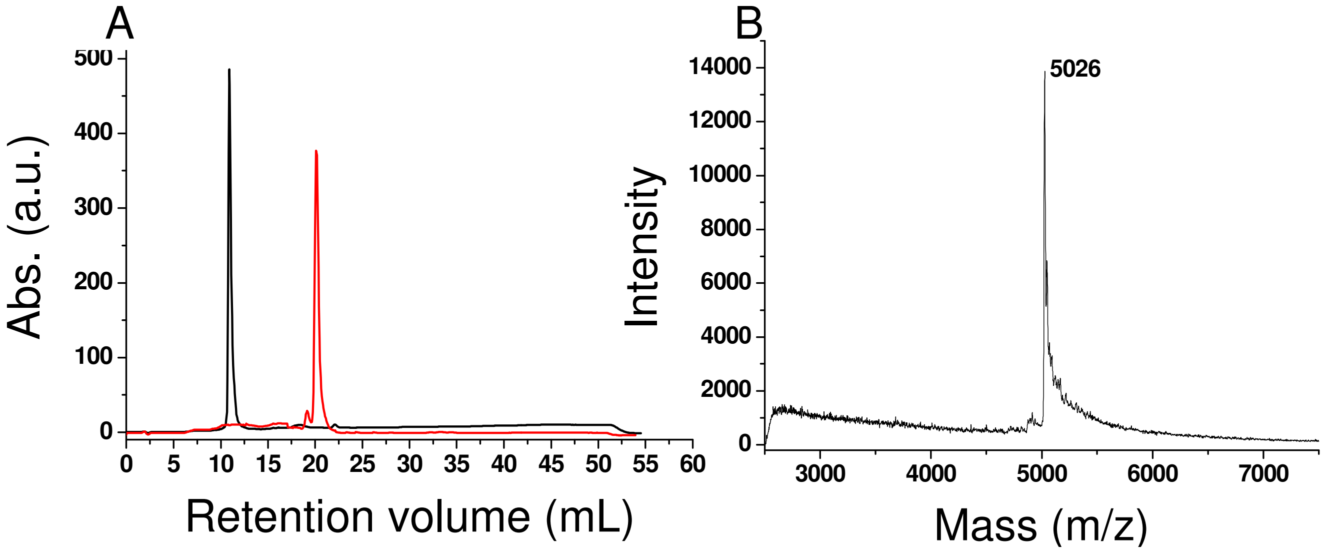


**Figure S4**. (**A**) Reverse phase HPLC chromatogram of purified $PPh_3$-labeled **L** probe (elution volume ~ 20 mL) and non-modified $NH_2$-C6-ODN (elution volume ~ 11 mL). Elution was monitored at 260 nm. **L** probe was analyzed by MALDI-TOF mass spectrometry. (**B**) MALDI-TOF mass spectrum of $PPh_3$-labeled **L** probe. Calculated: 5024; found: 5026.

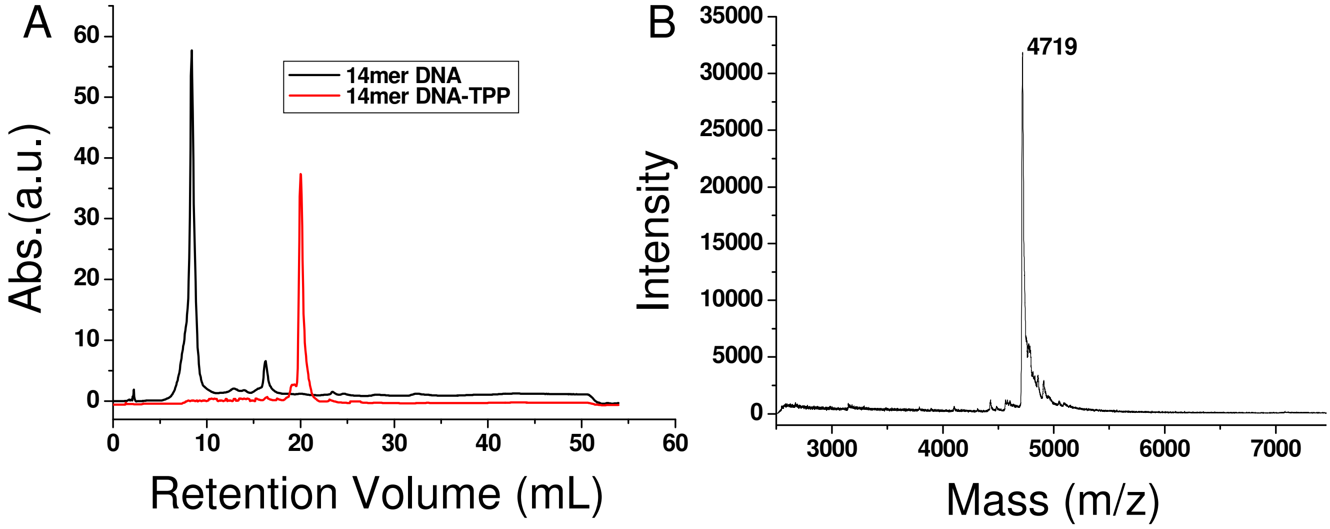


**Figure S5**. (**A**) Reverse phase HPLC chromatogram of purified $PPh_3$-labeled **R** probe (elution volume ~20 mL) and non-modified $NH_2$-C7-ODN (elution volume ~ 9 mL). Elution was monitored at 260 nm. **R** probe was analyzed by MALDI-TOF mass spectrometry. (**B**) MALDI-TOF mass spectrum of $PPh_3$-labeled **R** probe. Calculated: 4722; found: 4719.

## 8. Conditions for DNA-Templated Deiodination Reaction

A set of DNA-directed palladium catalyzed deiodination experiments were performed using varied concentration of target strand **T** ($C_T$ = 1 nM to 1 fM) in presence of 100 -1000 fold excess of iodo-BODIPY reporter molecules (**1** and **2)** under fixed concentration (1 μM) of probe and catalyst (10 μM $Na_2PdCl_4$ and 20 μM $NaBH_4$). DNA strands in NaOAc buffer (0.5 M, pH = 5.0) were mixed for 5 min in the presence of 75 mM NaCl solution and then heated up to 60 °C and cooled down slowly (1 °C / 1 min) to 24 °C using a thermal cycler. $Na_2PdCl_4$ solution in water was added to the hybridized DNA solution followed by aqueous solution of $NaBH_4$ under argon atmosphere and the reaction mixtures were shaken for additional 10 min followed by the addition of iodo-reporter dyes in water to initiate the catalytic reaction. The reaction mixtures, each with a final volume of 100 μL, were shaken for 4 h at 24 °C. Finally, visual color transition, UV/Vis and fluorescence spectroscopy were employed for characterization and quantification of the highly emissive dehalogenated products.

## 9. Kinetics of DNA-Templated Deiodination Reaction

The kinetics of the fluorogenic deiodination reactions were monitored using the following reaction conditions: pH = 5.0, 24 °C, 75 mM NaCl, 10 μM $Na_2PdCl_4$, 20 μM $NaBH_4$, 1 μM probe ODNs, 1 nM to 10 pM template and 10 nM monoiodo substrate **1**. As controls, the kinetics of the same conversion were also monitored without template or catalyst and with a single-base mismatch template. The fluorescence data were recorded on a SpectraMax M2 spectrophotometer (Molecular Devices, USA) using a 1 cm light-path quartz cuvette. The fluorescence signal was monitored every 15 sec at 510 nm (excitation: 500 nm).

## 10. Pd-Catalyzed Dehalogenation Assay in Presence of Crude Extract and Proteins

The DNA-mediated Pd-catalyzed dehalogenation reactions were carried out separately in presence of E.coli cell extract, DNA polymerase and BSA under identical reaction conditions (both probe and palladium concentrations were fixed at 1 μM, while the concentration of target and monoiodo substrate **1** were fixed at 100 pM and 1 nM, respectively).

In order to obtain bacterial crude cell extract, E.coli ER2738 was grown in LB medium until O. D. 600 = 1. Then the bacterial cells were broken down by the freeze/thaw method. The technique involves freezing a cell suspension in a liquid nitrogen bath and then thawing the cells at 37 °C. After this lysis step the insoluble fraction of the suspension was spinned down by centrifugation. The supernatant representing a clear cell extract was used as the medium for the dehalogenation assay. Both BSA and DNA polymerase were obtained from commercial sources.

The dehalogenation assay in presence of E.coli cell extract was carried out by mixing 50 μL of crude E.coli cell extract with DNA strands (**L**, **R** & **T**) in 50 μL of NaOAc buffer (0.5 M, pH = 5.0) containing 75 mM NaCl solution, 10 μM $Na_2PdCl_4$ and 20 μM $NaBH_4$ followed by heating to 60 °C and cooling down slowly (1 °C / 1 min) to 24 °C using a thermal cycler. Dehalogenation assays with DNA polymerase or BSA were carried out by mixing either 20 μL (2U/ μL) of DNA polymerase (40U ≈ 500 nM end concentration) or 20 μL of 100 μM BSA (end concentration = 20 μM) with DNA strands (**L**, **R** & **T**) in 80 μL of NaOAc buffer (0.5 M, pH = 5.0) containing 75 mM NaCl solution and 10 μM $Na_2PdCl_4$ and 20 μM $NaBH_4$ followed by heating to 60 °C and cooling down slowly (1 °C / 1 min) to 24 °C using a thermal cycler. Finally, the kinetics of all the dehalogenation reactions were monitored after addition

of 10 μL of monoiodo-reporter dye in water to the reaction mixture (final dye concentration 1 nM).

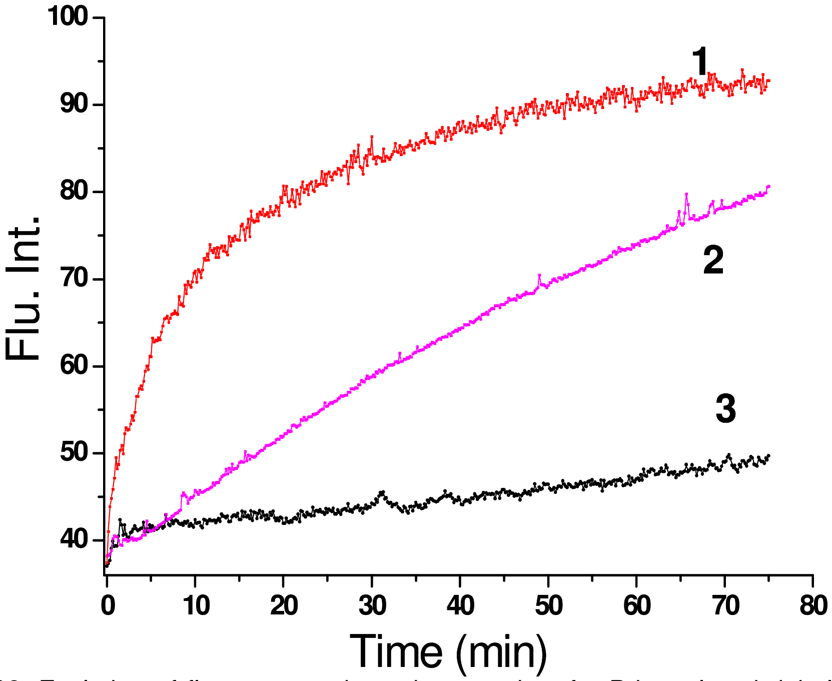


**Figure S6**. Evolution of fluorescence intensity over time for Pd-catalyzed dehalogenation assays in presence of bacterial cell extracts (curve 1), DNA polymerase (curve 2), and BSA (curve 3).

## 11. Determination of limit of detection

The detection limit of the DNA-templated catalyst for the fluorogenic conversion was calculated by a reported method.[2,3] We carried out a series of DNA-directed catalytic deiodination reactions with target ODN concentration of 1 fM to 1 pM under identical reaction conditions having equimolar concentrations of probe and Pd (10 μM) and at a fixed concentration of reporter molecules **1** or **2** of 500 fM. As a negative control, all reactions were also performed without template. It should be added here that the reaction conditions are kept constant in both cases, with and without template. The fluorescence intensity after reaction completion was measured for all reactions using a standard spectrophotometer and the resulting fluorescence intensities were plotted against template concentration. The limit of detection was determined to be the lowest measured concentration for which the mean fluorescence intensity exceeded that of the negative control by at least three standard deviations, $I_{control}$ (10 fM) = 0.16± 0.08; $I_{DNA}$ (10 fM) =2.64 ± 0.43 > $I_{control}$ (10 fM) + 3 × sd. Thus, 10 fM was determined to be the detection limit for DNA-templated dehalogenation that

uses monoiodo BODIPY **1** as precursor. However, the detection limit for fluorogenic conversion by using bisiodo BODIPY **2** as precursor was determined as 100 fM.

## 12. Determination of Quantitative Conversion Thresholds for **1** and **2**

Four sets of experiments were performed using mono- and bisiodo precursors to determine the quantitative threshold of complete conversion to fluorescent reporter dye. Both **1** and **2** were added in 30 -2000-fold excess to a range of target concentrations (1pM, 500 fM, 100fM and 10 fM) with a fixed amount of probe and catalyst. The lowest number of equivalents of precursor which could be completely converted to flurogenic product at a fixed target concentration was considered the threshold.

**Table S3**. Determination of quantitative conversion thresholds for **1** and **2**. Each threshold was determined by the complete conversion of the dye added in varied equivalents (from 30 to 1500X) at fixed $C_T$ = 10, 100, 500 and 1000 fM. √: complete conversion, **×**: incomplete conversion

| $T_C$ | $T_L$ & $T_R$ | monoiodo dye 1 | Total conv. 1 to 3 | bisiodo dye 2 | Total conv. 2 to 4 |
|---|---|---|---|---|---|
| 1000 fM | 1 µM | 2 nM (2000X) | × | 750 pM (750X) | × |
| | | 1 nM (1000X) | √ | 500 pM (500X) | √ |
| 500 fM | 1 µM | 750 pM (1500X) | × | 400 pM (800X) | × |
| | | 400 pM (800X) | √ | 100 PM (300X) | √ |
| 100 fM | 1 µM | 100 pM (1000X) | × | 10 pM (100X) | × |
| | | 50 pM (500X) | √ | 5 pM (50X) | √ |
| 10 fM | 1 µM | 500 fM (50X) | √ | 1 pM (100X) | × |
| | | 300 fM (30X) | √ | 100 fM (10X) | × |
| | | | | 10 fM (1X) | × |

### References


[1] U. Lauter, W.H. Meyer, V. Enkelmann, G. Wegner, *Macromol. Chem. Phys.* **1998**, *199*, 2129-2140.

[2] D. K. Prusty, A. Herrmann, *J. Am. Chem. Soc.* **2010**, *132*, 12197-12199.

[3] A. J. Baeumner, J. Pretz, S. Fang, *Anal. Chem.* **2004**, *76*, 888.
